# Case report: Upper gastrointestinal bleeding associated with pancreatic segmental portal hypertension: six case reports and literature review

**DOI:** 10.3389/fmed.2025.1522413

**Published:** 2025-02-19

**Authors:** Qian Miao, Zhongqing Zheng, Meiyu Piao, Hailong Cao, Bangmao Wang, Wentian Liu

**Affiliations:** Department of Gastroenterology and Hepatology, Tianjin Key Laboratory of Digestive Diseases, National Key Clinical Specialty, General Hospital, Tianjin Institute of Digestive Diseases, Tianjin Medical University, Tianjin, China

**Keywords:** pancreatic segmental portal hypertension, upper gastrointestinal bleeding, splenectomy, splenic artery embolization, case report

## Abstract

**Background:**

Pancreatic segmental portal hypertension (PSPH) is a clinical syndrome in which splenic vein hypertension is caused by obstruction, stenosis, or thrombosis of the splenic veins in the primary pancreatic disease. Gastrointestinal hemorrhage caused by gastric varices (GVs) is one of the life-threatening complications in the patients with left portal hypertension. The aim was to report our experience and discuss the manifestations, management, and prognosis of PSPH with upper gastrointestinal bleeding (UGIB).

**Method:**

We retrospectively analyzed six patients with PSPH and UGIB in our department. The clinical data were collected such as demographic information, medical history, and clinical presentation.

**Result:**

The autoimmune pancreatitis, pancreatic tumor, pancreatic surgery, chronic pancreatitis and pancreatic pseudocyst were diagnosed in six patients, respectively. Five patients presented with hematemesis and/or melena on admission, and one patient presented with fatigue. All patients had isolated GVs. Follow-up patients were treated with portal vein stenting in one case, laparoscopic splenectomy in two cases, endoscopic gastric fundic vein embolization and injection of Cyanoacrylate Glue in one case, and improvement in conservative treatment in two cases. All patients were alive at the last follow-up.

**Conclusion:**

PSPH should be seriously considered in patients with pancreatic disease with isolated GVs. It is particularly important to choose specific approaches for individual cases based on the primary disease, the severity of varicose veins and the general condition of the patients.

## 1 Introduction

Pancreatic segmental portal hypertension (PSPH) is a rare, localized, regional, elevated pressure in the left portal vein system due to non-hepatic causes (1). Unlike generalized portal hypertension (GPH), PSPH is not caused by systemic diseases such as cirrhosis and portal vein thrombosis, but stems from local factors related to the pancreas. Because the splenic vein is close to the dorsal side of the tail of the pancreatic body, this anatomical basis makes it possible for various pancreatic lesions to involve the splenic vein, resulting in the splenic vein being susceptible to thrombosis or being affected by exogenous compression, and eventually leading to splenic vein occlusion (2). After splenic vein obstruction, varicose blood vessels may form in all of these vessels. According to the literature, 45% ~ 72% of patients with PSPH develop upper gastrointestinal bleeding (UGIB) due to variceal rupture, and severe UGIB is often at fatal risk (3). The most common causes of PSPH and UGIB include acute and chronic pancreatitis and pancreatic cancer, and other less common causes include pancreatic tuberculosis, pancreatic abscess, pancreatic trauma, and pancreatic surgery (3, 4). Currently, splenectomy is the most common treatment for patients with PSPH who develop UGIB, However, for GPH, comprehensive treatment for the primary liver disease is often required. For patients with mild UGIB, refusal of surgery, or poor general condition, endoscopic therapy and splenic artery embolization (SAE) are the best options. However, endoscopic treatment carries the risk of rebleeding, and “post-infarct” syndromes such as abdominal pain, splenic abscess, sepsis, hematoma, and ectopic embolization are prone to occur after SAE (5). To date, there are no guidelines or consensus on the treatment of PSPH with UGIB. Therefore, we report six cases of PSPH complicated with UGIB caused by different pancreatic etiologies to share clinical experience and summarize its treatment in combination with relevant literature.

## 2 Case presentations

### 2.1 Case 1

**Figure 1:**
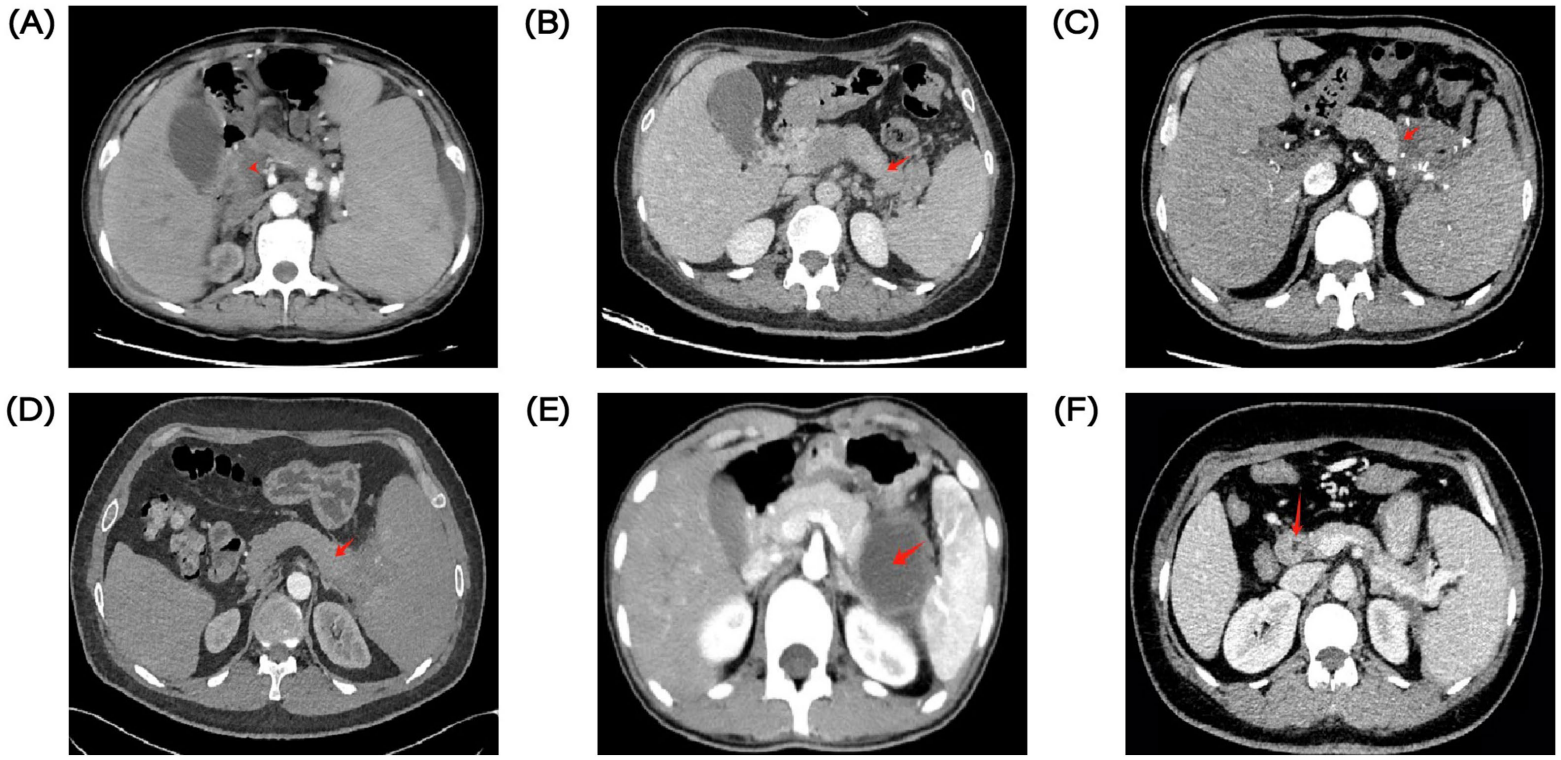
EGD images of varicose veins in 6 PSPH patients. **(A)** EGD image of case 1. **(B)** EGD image of case 2. **(C)** EGD image of case 3. **(D)** EGD image of case 4. **(E)** EGD images of case 5. **(F)** EGD images of case 6.

**Figure 2:**
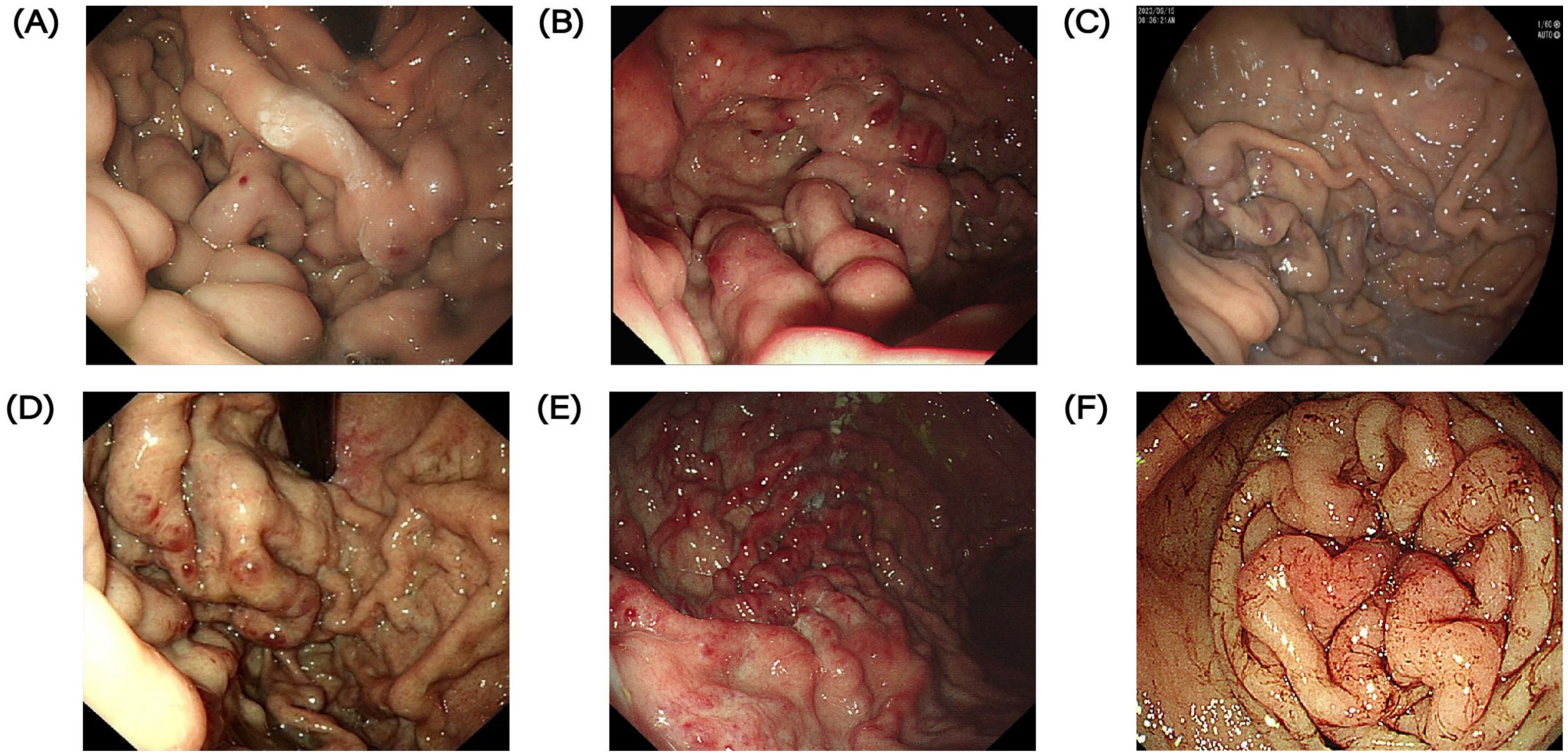
Contrast-enhanced CT images of the abdomen of pancreatic lesions in 6 patients with PSPH. Red arrows indicate pancreatic lesions. **(A)** Contrast-enhanced CT of the abdomen in case 1. **(B)** Contrast-enhanced CT of the abdomen in case 2. **(C)** Contrast-enhanced CT of the abdomen in case 3. **(D)** Contrast-enhanced CT of the abdomen in case 4. **(E)** Contrast-enhanced CT of the abdomen in case 5. **(F)** Contrast-enhanced CT of the abdomen in case 6.

A 51-year-old male was admitted to our department for presenting intermittent melena for 1 month. The patient had type II diabetes mellitus for 4 years. He underwent a needle biopsy for a pancreatic lump 5 years ago and was diagnosed as type II autoimmune pancreatitis. Afterwards, he was hospitalized three times for UGIB. Before admission, esophagogastroduodenoscopy (EGD) was performed and showed multiple varices with local red-color signs in the gastric fundus (Figure 1A). A contrast-enhanced abdominal CT scan demonstrated the presence of soft tissue nodules in pancreas head, splenomegaly, widening of the portal vein with diameter of approximately 17 mm, and aggravation of portal hypertension (Figure 2A). After administration of intravenous proton pump inhibitor (PPI) and octreotide, the patient’s condition was improved. Given that history of recurrent UGIB, the patient was transferred to department of General Surgery and underwent portal angiography. No images were obtained from the portal vein main trunk to the portal vein and the portal vein adduction part of the superior mesenteric vein. Simple splenectomy alone could not solve the portal occlusion, so balloon dilation and splenic vein stent implantation (SVS) was performed. There was no evidence of bleeding during follow-up.

### 2.2 Case 2

A 51-year-old female was admitted to our hospital due to melena for 1 month and hematemesis for 10 days. EGD demonstrated severe varicose veins in the fundus and body of the stomach (Figure 1B). Positron emission tomography-computed tomography (PET-CT) showed a mass in the pancreatic tail with a high probability of malignancy, splenomegaly, and high possibility of tumor thrombus involving portal vein trunk and splenic veins. We suggested the patient underwent a needle biopsy, she refused and was discharged. Later, she was diagnosed with a pancreatic neuroendocrine tumor in another hospital and received targeted therapy. After 1 year, she visited our department with complaints of hematemesis for 1 day. A contrast-enhanced abdominal CT scan demonstrated tumorous lesions of pancreatic origin, the tumor invaded the splenic artery, splenic vein, portal vein, superior mesenteric vein and with hepatic metastasis, tumor thrombus enhancement in portal vein, and portal hypertension (Figure 2B). Obviously, her cancer continued to progress with an increased number of metastatic lesions. Her bleeding was controlled after fasting, blood transfusion, and aggressive acid suppression. No further treatment was attempted, such as pancreatectomy and splenectomy, in view of the advanced disease. After 2 years, the patient was admitted again for hematochezia. Endoscopic examination showed significant gastric varices with red signs compared to before, with a maximum diameter of 1 cm.

### 2.3 Case 3

A 61-year-old male was admitted to our department with intermittent melena for 3 month. The patient had a history of recurrent UGIB and had type II diabetes mellitus for 3 months. He underwent laparoscopic resection of the pancreatic body and tail lesions 6 months before admission for pancreatic borderline tumor. EGD revealed GVs with blue and local red-color signs (Figure 1C). A contrast-enhanced abdominal CT scan demonstrated splenomegaly, low density intra-splenic nodular shadow, multiple tortuous vascular shadows around the spleen (Figure 2C). After administration of acid suppression and pancreatic enzyme secretion reduction, there was no signs of rebleeding during hospitalization. Later, he transferred to department of General Surgery for laparoscopic total splenectomy and pericardial devascularization. The patient had splenomegaly and severe anemia, and significant pericardial varices. According to the condition, he transferred to department of General Surgery for laparoscopic total splenectomy and pericardial devascularization.

### 2.4 Case 4

A 41-year-old male was admitted to our department with complaints of melena for 6 days. He underwent EGD which demonstrated a mass of severe varices in the gastric fundus with a red color sign and duodenal ulcer (Figure 1D). A contrast-enhanced abdominal CT scan showed an irregular soft tissue density mass in the pancreatic tail-Spleen hilum, with splenomegaly (Figure 2D). The patient showed increased abnormal metabolism of the mass on PET-CT. He was transferred to department of General Surgery. The pancreatic tail mass can also be seen in the surgery, which involved the splenic portal vessels. After comprehensive consideration, laparoscopic-assisted partial pancreatic resection, splenectomy and pericardial devascularization was performed. Diagnosis of chronic fibrous pancreatitis was considered based on postoperative pathological findings. There was no evidence of rebleeding during follow-up.

### 2.5 Case 5

A 20-year-old male was admitted to our department with abdominal pain for more than 1 month and intermittent coffee-ground vomiting twice. 1 month ago, the patient underwent laparoscopic partial pancreatic resection for pancreatic solid pseudopapillary tumors. EGD demonstrated varicose veins in the gastric fundus with a red color sign (Figure 1E). A contrast-enhanced abdominal CT scan showed pancreatic cystic lesion in the tail of pancreas, and splenomegaly (Figure 2E). After treatment with rehydration and acid suppression, his condition improved. There was no sign of rebleeding during hospitalization and follow-up.

### 2.6 Case 6

A 40-year-old woman is admitted to hospital with fatigue for 2 years. The patient had type II diabetes for 5 years and hypothyroidism for 4 months. 1 year ago, bone penetration in the outer courtyard was active, and bone marrow hyperplasia was active, but there were no abnormalities in autoimmune liver, IgG4 and other related laboratory tests. He was admitted to the hospital for EGD, which revealed a large amount of dark red blood in the stomach, several variceal veins with a diameter of about 0.5 cm were seen in the gastric fundus, and variceal veins were seen on the side of the large curvature of the gastric fundus junction, with a size of about 1.5*2.0 cm, and the surface was red (Figure 1F). CT of thoracic and abdominal vessels: the portal vein is thickened with a diameter of about 15 mm. With varices in the fundus, around the proximal corpus, and in the hilar region of the spleen. CT abdomen: slightly thickened portal vein, thickened perigastric and splenic hilar blood vessels, splenomegaly; Pancreatic pseudocyst (Figure 2F).

## 3 Results

We included six patients treated in our department between July 2022 and August 2024 who were considered to have a diagnosis of PSPH with UGIB. At present, no unified diagnostic criteria for PSPH have been established at home and abroad. In this study, the diagnosis of PSPH with gastrointestinal bleeding is mainly based on the patient’s clinical manifestations, CT, gastroscopy and other auxiliary examinations: 1. The patient has pancreatitis, pancreatic tumor and other pancreatic diseases, and has gastrointestinal bleeding manifestations such as hematemesis and hematochezia. 2. Pancreatic lesions, portal vein thickening, varicosities of the fundic veins and splenic vein thrombosis are seen on CT, while the liver is morphologically, size-wise, and texture-wise normal. 3. Isolated gastric varices diagnosed by gastroscopy, ultrasonography and gastroscopy. 4. Exclude portal hypertension due to cirrhosis.

Overall, six patients with PSPH and UGIB were enrolled. There were four males and two females. The average age was 44 ± 14.05 years (minimum 20 years, maximum 61 years). The six patients had different etiologies, one had chronic fibrous pancreatitis, one had type II autoimmune pancreatitis, one was diagnosed as pancreatic neuroendocrine tumor with multiple metastases, two had Pancreas Surgery, and one of them had concomitant pancreatic pseudocyst. Most patients had hematemesis and/or melena as first symptom, only one patient complained of fatigue, and two patients were accompanied by abdominal pain. Each patient presented with isolated GVs on EGD. All patients underwent CT, and had no liver diseases such as liver cirrhosis. One patient demonstrated a presence of tumor thrombus in the portal vein and splenic vein.

The average hemoglobin and albumin levels of all patients were low, with average values of 66 g/L and 33 g/L, respectively. There was no significant increase in liver function and indicators such as amylase and lipase.

One patient had visible bleeding under endoscopy and underwent gastric vein embolization and injection of Cyanoacrylate Glue. The remaining patients did not show active bleeding under endoscopy, so no endoscopic treatment was performed, and all were given conservative treatment mainly to inhibit acid and pancreatic enzyme secretion. Three patients were later transferred to the general surgery department for further treatment, one of whom underwent splenic vein stent implantation, and two underwent laparoscopic splenectomy and pericardial vascularization. Five patients did not experience UGIB during the follow-up period. The demographic and clinical characteristics of the patients are summarized in Table 1.

**Table 1:** Characteristics of the patients with pancreatic portal hypertension.

Variables	Case 1	Case 2	Case 3	Case 4	Case 5	Case 6
Age (years)	51	51	61	41	20	40
Gender	Male	Female	Male	Male	Male	Female
Smoking or not	No	Yes	Yes	Yes	No	No
Drinking alcohol or not	No	Yes	No	No	No	No
Pancreatic diseases	Type II autoimmune pancreatitis	Pancreatic neuroendocrine tumors (T4N0M1a Stage IV)	After pancreatic surgery (T2N0M0 Stage IB)	Chronic fibrous pancreatitis	After pancreatic surgery combined with pancreatic pseudocyst (T2N0M0 Stage IB)	Pancreatic pseudocyst
Clinical presentation	Black stools, fatigue	Black stools, Hematemesis, abdominal pain	Black stools, dizzy	Black stools	Hematemesis, abdominal pain	Fatigue
Comorbidities	Type II diabetes	Hypertension grade 1	Type II diabetes	-	-	Type II diabetes、Hypothyroidism
EGD	Gastric varicose veins	Gastric fundus, corpus varices	Gastric varicose veins	Gastric varicose veins	Gastric varicose veins	Gastric varicose veins
Sarin classification	Type I IGV	Type I IGV	Type I IGV	Type I IGV	Type I IGV	Type I IGV
LDRf classification	LgfD0.3 Rf1	LgfD0.3 Rf1	LgfD1 Rf1	LgfD1 Rf1	LgfD0.3 Rf1	LgfD0.3 Rf1
WBC (*10^9/L)	2.70	2.33	3.61	2.75	5.04	2.02
RBC (*10^12/L)	1.29	3.39	2.07	2.87	2.87	2.82
PLT (*10^9/L)	81	152	110	70	235	139
HB (g/L)	30	97	55	69	86	62
ALB (g/L)	27	33	34	30	35	41
CRP (mg/dL)	0.49	0.18	0.59	12.00	0.33	-
D-dimer (ng/mL)	1,237	1784	671	2,680	2,726	213
TBIL (umol/L)	41.4	2.0	8.8	19.9	6.1	45.4
DBIL (umol/L)	14.5	-	3.2	6.6	2.1	8.5
ALT (U/L)	7	10	7	3	25	9
AST (U/L)	17	16	10	6	12	14
ALKP (U/L)	38	56	71	59	40	53
GGT (U/L)	6	17	12	17	16	10
AMY (U/L)	63	93	59 (Urinary)	61 (Urinary)	81	-
LIPA (U/L)	147	365	-	-	363	-
Types of treatment	SVS	Conservative treatment	Laparoscopic total splenectomy and pericardia vascular dissection	Laparoscopic assisted partial pancreatectomy, splenectomy, and pericardia vascular dissection	Conservative treatment	Endoscopic ultrasound fundic vein embolization, injection of Cyanoacrylate Glue
Outcomes	Survived	Survived	Survived	Survived	Survived	Survived

EGD, esophagogastroduodenoscopy; WBC, white blood cell; RBC, red blood cell; PLT, platelet; HB, hemoglobin; ALB, albumin; CRP, C-reactive protein; TBIL, total bilirubin; DBIL, direct bilirubin; ALT, alanine aminotransaminase; AST, aspartate aminotransferase; ALKP, alkaline phosphatase; GGT, γ-glutamyl transpeptidase; AMY, amylase; LIPA, lipase; SVS, splenic vein stent implantation. The reference range of laboratory tests: WBC: 3.50~9.50*10^9/L; RBC: 4.30~5.80*10^12/L; PLT: 125~350*10^9/L; HB: 130~175 g/L (male), 115~150 g/L (female); ALB: 40~55 g/L; CRP: <0.08 mg/dL; D-dimer: 0 ~ 500 ng/mL; TBIL: ≤26umol/L; DBIL: 0.0~7.0umol/L; ALT: 0.0~7.0umol/L; AST: 15~40 U/L; ALKP: 15~40 U/L; GGT: 10~60 U/L; AMY: 30~110 U/L; LIPA: 23~300 U/L.

## 4 Discussion

PSPH, also known as Left-Sided Portal Hypertension (LSPH), is a type of extrahepatic portal hypertension, accounting for 5 percent of extrahepatic portal hypertension (6), which is caused by the spleen vein being compressed by a mass or cyst, or blocked by thrombosis, making it difficult for blood from the spleen to flow through the short gastric vein to the stomach and eventually to the portal vein, but instead to the collateral circulation of the short gastric vein, resulting in GVs (3, 7). Isolated GVs is a clinical feature of PSPH, but GVs of PSPH may also be secondary to esophageal variceal rupture or portal hypertension gastropathy (8). Although the clinical incidence of PSPH complicated by UGIB is low, the associated GVs still have a high risk of mortality from ruptured bleeding.

When UGIB was caused by PSPH, hemodynamics should be optimized by injection of crystals or colloids and blood transfusions, in addition, vasoconstrictors (e.g., somatostatin, vasopressin), acid-suppressing drugs (e.g., PPIs, H2 receptor antagonists), and three-lumen two-cyst tubes should be used to stop the bleeding urgently. When the patient’s vital signs are stabilized, it is also necessary to address the root cause of hypertension on the splenic side of the portal venous circulation and eliminate the risk of recurrent bleeding combined with the patient’s primary disease (8, 9).

Unlike PSPH, GPH refers to a pathologic condition in which pressure within the portal system is generally elevated, usually caused by systemic disease inside and outside the liver, and involves the entire portal system, rather than being confined to a small segment. For this reason, its diagnosis usually requires evaluation of the entire portal venous system by ultrasonography, CT, endoscopy, and other multifaceted examinations. In terms of treatment, both need to be treated for the cause, elective transjugular intrahepatic portosystemic shunt (TIPS), and liver transplantation are often used for GPH (10). Taken together, GPH has a wider range of effects, which requires a comprehensive assessment of portal venous system involvement and comprehensive treatment measures for the underlying disease, while PSPH focuses more on the assessment and management of local pancreatic diseases.

Splenectomy is often recommended for patients with PSPH and UGIB (11, 12), it can not only reduce gastric venous congestion due to blood flow into the collateral circulation of the short gastric veins by removing the entire spleen to terminate the blood supply to the splenic artery, but also allows surgeons to treat possible pancreatic disease concurrently (13). However, in recent years, the disadvantages of splenectomy have also been frequently criticized. First, splenectomy, as an open procedure requiring general anesthesia, carries certain anesthetic and surgical risks, and a mortality rate of up to 8% has been reported with splenectomy (14). Second, pancreatic diseases such as pancreatitis and pancreatic tumors are the most common causes of PSPH (3, 15). However, surgery is often difficult in difficult settings due to inflammatory adhesion due to pancreatitis or other complications, particularly during acute exacerbations (16). At the same time, splenectomy results in a loss of spleen immunity, which may lead to overwhelming post-splenectomy infection (OPSI), also known as post splenectomy syndrome, with a mortality rate of up to 50% (17). Despite these risks, splenectomy is used as the mainstay in treatment of PSPH with UGIB because it minimizes blood flow to gastric and esophageal varices. In a meta-analysis, Liu et al. (18) concluded that splenectomy was more effective in reducing the incidence and mortality of UGIB than other treatment strategies.

In addition, prophylactic splenectomy is generally not recommended for patients with PSPH who have not yet experienced UGIB due to the insignificant differences in overall survival (19), the presence of biliary digestive anastomotic vein appendages (20), and avoidable complete loss of spleen function.

Based on the risks associated with splenectomy, SAE may be an alternative treatment. It works the same as splenectomy and can prevent bleeding by reducing blood flow from the spleen to the collateral veins (3), thereby decompressing the associated varices. The most important advantages of SAE over splenectomy are its minimally invasive nature and preservation of spleen function, and it has been shown that SAE is an effective method for UGIB caused by PSPH (21), but it may still cause some complications, including splenic abscess, sepsis, and splenic rupture (5). In general, SAE is the embolization of 50 to 70% of the splenic parenchyma (22), and when embolization of too much of the spleen parenchyma, more and more serious complications may arise (23).

The two-step embolization method and modified endoscopic ultrasound (EUS)-guided selective N-butyl-2-cyanoacrylate (NBC) injection have shown greater advantages in minimizing complications associated with SAE. When a large portion of the spleen is completely embolized in a single operation, postembolic syndrome may occur, presenting with fever, abdominal pain, etc. (24). Therefore, some patients may require a two-step embolization method. Wang et al. (25) suggested that partial splenic embolization can be performed to achieve infarction of 60 to 70% of the splenic volume in patients with PSPH and UGIB after their vital signs have stabilized, and a second embolization treatment was performed a month later. Patients treated with the two-step embolization method experienced a reduction in symptoms of postoperative fever and abdominal pain compared with those treated with one-step embolization, and in addition, the risk of pseudoaneurysms was reduced with this approach (26). Modified EUS-guided selective NBC injection therapy has also been widely used due to its outstanding advantages, and compared with traditional endoscopic injection, modified EUS-guided NBC injection has performed better in terms of reduced NBC dose, fewer complications, less risk of additional radiation exposure, and lower cost of endoscopic procedures (27–29). However, the disadvantage is that it is time-consuming and requires more technical skills from the operator (29). In addition, SAE can also be used as a preoperative management for splenectomy to reduce intraoperative blood loss and conversion to open surgery (30, 31). Liu (18) and Fujitani RM et al. (32) suggest that SAE-assisted splenectomy is more appropriate in high-risk patients and can significantly reduce intraoperative blood loss and operative time.

Unlike the two methods described above, SVS relieves pressure on collateral circulation by placing a stent in the splenic vein to restore blood flow to the collateral circulation (33), thus restoring normal blood circulation while completely preserving the spleen, thereby avoiding complications associated with loss of spleen function (34). SVS has been reported to be effective in the treatment of UGIB caused by PSPH in many previous case studies (30, 35–37). In a retrospective study of the effects of splenectomy and SVS on treatment of PSPH, Liu et al. (38) found that SVS was associated with a lower incidence of surgical complications such as bleeding and infection, a shorter hospital stay, and a lower overall cost of care compared with splenectomy. In another retrospective study comparing SAEs and SVS, Bo Wei et al. (33) noted that patients who received SVS (7.1%) had a significantly lower rate of rebleeding than those who received SAE (47.8%), and that SVS had a more significant advantage in preventing PSPH rebleeding. However, according to previous studies, the success rate of SVS is only 53.8% (33), and its use may be limited by a low success rate, and stent passage is difficult in some sites of severe splenic vein occlusion, particularly long splenic vein occlusion due to chronic pancreatitis, which is a major cause of SVS failure (33). In cases where it is difficult to open a stenosis segment of the splenic vein by portal vein approach, the addition of a splenic venous puncture method may increase the probability of reopening of the splenic vein occlusion segment (37).

In conclusion, the treatment of PSPH with gastrointestinal bleeding is divided into treatment of the primary disease of the pancreas and management of gastrointestinal bleeding. Splenectomy, splenic artery embolization, splenic vein stenting are all effective for gastrointestinal bleeding due to PSPH. Among these, splenectomy is most widely used because it minimizes blood flow to the stomach and simultaneously treats the primary disease of the pancreas. When the patient has recurrent pancreatitis causing tissue adhesion and it is difficult to perform surgery, splenic artery embolization can be used as an alternative, however its application is limited due to its possible complications such as splenic rupture and septicemia. Splenic vein stent implantation has the advantages of minimal invasion, preservation of spleen and fewer postoperative complications, but has certain technical requirements for the operator. When the patient is suffering from splenic vein occlusion due to chronic pancreatitis, the operation is difficult to carry out. A total of 3 patients were treated surgically in this study. Splenic vein, portal vein stent implantation in one patient for obstruction of portal, splenic, and superior mesenteric venous junctions. One patient had a pancreatic space-occupying lesion involving the spleen, and was selected for pancreatic lesion resection and splenectomy. Another one patient underwent splenectomy for massive splenosis and severe anemia. It can be seen that these patients have their own characteristics, and blind selection of splenectomy is not advisable, individualized treatment is particularly important.

In this study, six patients with pancreatic disease and UGIB were collected. The mean hemoglobin and albumin levels in six patients were low, which may be related to long-term chronic gastrointestinal blood loss. Five patients complained of melena and hematemesis, and were able to receive timely treatment. The patient in case 6 started with fatigue, and white cells, red blood cells and platelets were low. Complete abdominal CT showed pancreatic pseudocyst and portal hypertension. Gastroscopy shows dark red blood in the fundus and several varicose veins. After finding no contraindications, we performed ultrasound gastroscopic venous embolization combined with injection of Cyanoacrylate Glue, and the patient recovered well after surgery. This case is enough to remind us that for patients without typical UGIB manifestations, we should pay attention to routine blood count, abdominal CT and gastroscopic findings.

The etiology and treatment methods of the six patients in this study are abundant, but due to the small sample size, this study is only descriptive statistics, and a large sample study is needed to summarize the clinical characteristics of such patients in the future, so as to make timely diagnosis and treatment.

Since the initiating factors of PSPH are various types of primary pancreatic diseases, it is necessary to pay more attention to the primary pancreatic diseases while solving the problem of UGIB, and take corresponding treatment to achieve the purpose of effectively controlling the symptoms and alleviating the progression of the disease.

## 5 Conclusion

In conclusion, for patients with PSPH and UGIB, treatment should be individualized for their primary pancreatic disease. In this study, all six patients with PSPH and UGIB were treated with SVS, splenectomy, and treatment for primary pancreatic disease according to their actual conditions. In this study, we summarized the patients diagnosed with PSPH complicated with UGIB in our department and summarized the current mainstream clinical methods for the treatment of PSPH with UGIB, in order to provide reference opinions for the diagnosis, individualized treatment and prevention of PSPH complicated with UGIB.

## Data Availability

The original contributions presented in the study are included in the article/supplementary material, further inquiries can be directed to the corresponding author.
